# Best pain management for critical older patients in the surgical ICU

**DOI:** 10.1186/cc14570

**Published:** 2015-03-16

**Authors:** W Huang

**Affiliations:** 1 Mackay Memorial Hospital, Taipei, Taiwan

## Introduction

To determine which of three methods of pain management provided the best pain control in severe ASA III older patients in the surgical ICU (SICU). As technology improves, more older patients benefit from surgery and need SICU care. Older surgery patients frequently present two medical problems. First is unspecific symptoms and decreased pain sensation resulting in delayed diagnosis. Second, they usually are not given enough perioperative pain relief. Optimal pain management results in perioperative stable hemodynamic status and decreased morbidity, mortality, length of stay and medical costs.

## Methods

A retrospective cohort study chart review of 1,872 all-cause patients in a 16-bed SICU during April 2011 to September 2012. Unconsciousness, uncooperative, ASA <III and <65-year-old patients were excluded. The primary point was to compare effectiveness of three different methods of pain management: P.R.N. i.v. Demerol/NSAID (D/N), i.v. patient-controlled analgesia (PCA) and patient-controlled epidural analgesia (PCEA), in three different conditions: rest, movement and coughing, with visual analogue scales (VAS 0 to 100). Secondary point was patient satisfaction.

## Results

A total of 1,292 patients were excluded. VAS results are presented in Table 1 as mean ± SD. At rest, the PCEA group is significantly better than the other two groups. When at movement, there is no difference between the PCEA group and the D/N group but both are better than the PCA group. While coughing, the PCEA group is worse than the D/N group, although there is no difference between the PCEA group and the PCA group. The PCEA group gets the best grades in patient satisfaction.

**Table 1:** 

	Demerol/NSAID (*n = *449)	PCA (*n = *62)	PCEA (*n = *69)	*P *value
VAS (rest)	21 ± 14 *	19 ± 9*	12 ± 10	<0.001
VAS (movement)	35 ± 20	41 ± 12*	33 ± 11	0.02
VAS (coughing)	40 ± 24*	58 ± 14	51 ± 14	<0.001

*Significant difference.

## Conclusion

PCEA provided better pain control at rest than the other two methods, whereas P.R.N. Demerol/NSAID and PCEA were somewhat better than PCA when patients were moving. While coughing, P.R.N. Demerol/NSAID provided the best pain control. However, patient satisfaction was significantly better with PCEA.

